# Tryptophan and arginine catabolic enzymes and regulatory cytokines in clinically isolated syndrome and multiple sclerosis

**DOI:** 10.1002/cti2.1037

**Published:** 2018-08-16

**Authors:** Lilian Cha, Anderson P Jones, Stephanie Trend, Scott N Byrne, Marzena J Fabis‐Pedrini, William M Carroll, Robyn M Lucas, Judith M Cole, David R Booth, Allan G Kermode, Prue H Hart

**Affiliations:** ^1^ Telethon Kids Institute University of Western Australia Perth WA Australia; ^2^ Faculty of Medicine and Health Westmead Institute for Medical Research University of Sydney Westmead NSW Australia; ^3^ Centre for Neuromuscular and Neurological Disorders Perron Institute for Neurological and Translational Science Sir Charles Gairdner Hospital University of Western Australia Perth WA Australia; ^4^ National Centre for Epidemiology and Population Health Research School of Population Health Australian National University Canberra ACT Australia; ^5^ St John of God Dermatology Subiaco WA Australia; ^6^ Institute for Immunology and Infectious Disease Murdoch University Perth WA Australia

**Keywords:** 3‐dioxygenase, arginase, clinically isolated syndrome, cytokines, indoleamine 2, multiple sclerosis, peripheral blood mononuclear cells

## Abstract

**Objectives:**

Clinically isolated syndrome (CIS) is the earliest clinical episode in multiple sclerosis (MS). A study of circulating cells from patients with CIS may help us understand the transition to, and processes associated with, the development of MS.

**Methods:**

As immune cell activity can be determined by flux through metabolic pathways, the mRNA expression of l‐tryptophan‐ and l‐arginine‐catabolising enzymes, indoleamine 2,3‐dioxygenase (IDO) 1 and IDO2 and arginase (ARG) 1 and ARG2, respectively, was compared between peripheral blood mononuclear cells (PBMCs) from healthy controls, and patients with CIS and definite MS. As one measure of cell function, cytokine mRNA levels were analysed directly *ex vivo* and in cells after culture for 4 h in the absence of regulatory factors in autologous serum.

**Results:**

When measured directly *ex vivo*, the expression of IDO and ARG was greater in cells from patients with CIS and MS than cells from healthy controls. Although not linked to IDO and ARG expression, PBMCs from the CIS patients were characterised by low IL‐10 and TGFB mRNA levels and not by greater expression of proinflammatory cytokines. When the cells were cultured for 4 h without autologous serum, pro‐ and anti‐inflammatory cytokine mRNA levels positively correlated with IDO1 expression, and TGFB mRNA levels correlated with ARG1 expression.

**Conclusion:**

Higher IDO and ARG expression in CIS and MS provides one sustained homeostatic mechanism to control MS‐associated inflammation. However, potent extrinsic mediators in serum may regulate immune cell function in CIS and associations between IDO, ARG and cytokine expression.

## Introduction

The survival and activity of immune cells are determined in large part by flux through metabolic pathways, including those providing ATP interlinked with those providing the amino acids and nucleotides for protein and nucleic acid synthesis.^1^ For example, increasing l‐arginine (Arg) in activated T cells induces global metabolic changes including a shift from glycolysis to oxidative phosphorylation.^2^ Immune cells require amino acids for proliferation, and production of proinflammatory mediators. In turn, increased expression of the enzymes that catabolise amino acids such as l‐tryptophan (Trp) and l‐arginine (Arg) could limit the activity of inflammatory immune cells and restrict inappropriate autoimmune responses. Increased expression of these enzymes may not only reduce the supply of amino acids that limit immune cell survival, activation and expansion, but they would allow the production of multiple downstream metabolites such as the kynurenines and spermidine with proven immunomodulatory properties.^3^, ^4^, ^5^

The first and rate‐limiting enzymes in haematopoietic cells responsible for Trp metabolism are indoleamine 2,3‐dioxygenase (IDO)1 and IDO2. The third enzyme that catabolises Trp, tryptophan 2,3‐dioxygenase, is expressed mainly in the liver. Increased IDO expression associates with tolerogenic pathways because of Trp depletion and has been associated with the formation of tolerogenic dendritic cells and induction of T regulatory (T_reg_) cells.^6^, ^7^ Kynurenines are Trp metabolites that stimulate aryl hydrocarbon receptors and associated downstream immunoregulatory pathways.^8^, ^9^

Two arginases, arginase 1 and arginase 2 (ARG1 and ARG2), expressed by different genes catabolise Arg in myeloid cells (macrophages, dendritic cells) and activated T cells. ARG1 is principally cytosolic whilst ARG2 is expressed in mitochondria. ARG1 and ARG2 convert Arg to l‐ornithine and urea; the former molecule is a precursor for glutamic acid, proline and polyamine synthesis and helps to create an anti‐inflammatory environment conducive to tissue repair. Arg is also catabolised by a third enzyme, inducible NO synthase (NOS2), to produce NO and l‐citrulline. NO enhances the differentiation of monocytes to ‘classically activated’ macrophages whilst increased ARG1 expression has been linked with prohomeostatic ‘alternatively activated’ macrophages that do not produce NO.^10^, ^11^ Arg is required for the production of TNF‐α.^12^, ^13^ In T cells, ARG expression, by reducing Arg levels, reduces the metabolic fitness of the cells and reduces the generation of central memory‐like cells.^2^

In human peripheral blood cells from healthy individuals, approximately 70% of mRNA for IDO1 and ARG1 are expressed by CD14^+^ cells, 15% by CD4^+^ T cells, 10% by CD8^+^ T cells and 5% by B cells.^14^

A recent study from Argentina of patients with relapsing–remitting MS (mean duration of disease of 5.2 years) suggested that circulating blood cells of MS patients expressed reduced levels of IDO1 and ARG1.^14^ This would provide a proinflammatory environment with increased availability of Trp and Arg for the Th1‐ and Th17‐driven autoimmune responses associated with MS pathogenesis.^15^ In the study reported here, the focus was on expression of IDO and ARG during the MS disease course. PBMCs were studied from individuals with clinically isolated syndrome (CIS), the earliest clinical episode in MS, but their brain magnetic resonance imaging (MRI) results do not fulfil all criteria for diagnosis as MS. Levels of IDO and ARG mRNA were compared with those of cells from healthy controls (HCs), as well as patients with established MS. The aim was to determine whether changes in catabolic enzyme expression were a characteristic of the early form of disease, or reflected a later development in established MS. The effect of a potential immunomodulatory treatment, namely narrowband UVB phototherapy (311–312 nm),^16^, ^17^ on the expression of IDO and ARG by PBMCs was also examined. Associations between IDO and ARG expression and the function of the PBMCs were assessed by analysis of mRNA levels of genes encoding pro‐ and anti‐inflammatory cytokines. The effect of intrinsic or extrinsic determinants of IDO and ARG expression was examined by culture of PBMCs for 4 h in the absence of regulatory factors in autologous serum. This study identified changes in PBMCs from CIS patients that suggest lower expression of anti‐inflammatory cytokines precedes the expression of proinflammatory cytokines in the development of MS.

## Results

### mRNA levels of IDO1 and IDO2, and ARG1 and ARG2, are higher in cells from MS patients

**Figure 1:**
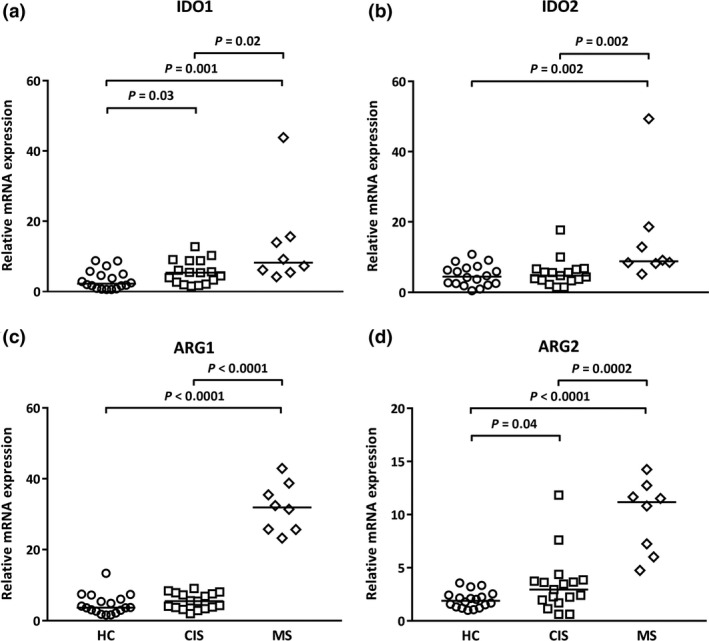
Higher mRNA levels of IDO and ARG in PBMCs from CIS and MS patients. **(a)** IDO1, **(b)** IDO2, **(c)** ARG1 and **(d)** ARG2. Data for each sample are expressed relative to UBE2D2 mRNA. The median (horizontal bar) and significant differences between expression in PBMCs from HCs (*n* = 18, circles), CIS (*n* = 17, squares) and MS patients (*n* = 8, diamonds) are indicated.

To establish whether there were differences in the expression of genes for IDO and ARG in PBMCs from HCs and CIS and MS patients, relative mRNA expression (normalised to ubiquitin‐conjugating enzyme E2 D2 [UBE2D2]) was compared. There were significantly higher levels of IDO1 and ARG2 mRNA in PBMCs from CIS patients relative to HCs. mRNA levels for all enzymes were significantly elevated further in cells from MS patients (Figure 1).

### The expression of the anti‐inflammatory cytokines, IL‐10 and TGFB, is lower in CIS

**Figure 2:**
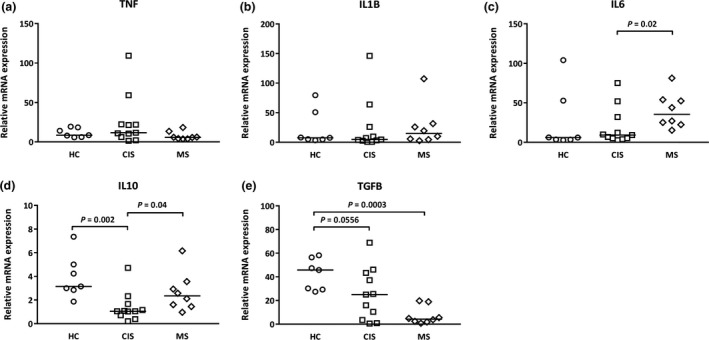
Altered IL‐6, IL‐10 and TGFB levels in PBMCs from CIS and MS patients. **(a)** TNF,** (b)** IL‐1B, **(c)** IL‐6, **(d)** IL‐10 and **(e)** TGFB. Data for each sample are expressed relative to UBE2D2. The median (horizontal bar) and significant differences between expression in PBMCs from HCs (*n* = 7, circles), CIS (*n* = 11, squares) and MS patients (*n* = 8, diamonds) are indicated.

As amino acid catabolism can modulate cytokine production and immune cell activity^5^, levels of mRNA for cytokine genes were assessed. Greater IL‐6 mRNA levels were detected in PBMCs from MS compared with CIS patients; however, no significant differences were observed for TNF or IL‐1B expression between the HC, CIS and MS groups (Figure 2). In contrast, in comparison with cells from HCs and MS patients, there was significantly less IL‐10 mRNA expression in cells from patients with CIS. There was significantly lower TGFB expression in cells from both CIS and MS patients compared with cells from HCs (Figure 2).

When correlations between the expression of the proinflammatory and anti‐inflammatory cytokines and IDO and ARG were analysed, no associations were observed in PBMCs from the HC, CIS and MS groups, with the exception of a positive correlation between ARG1 and TGFB mRNA in cells from CIS patients (columns for PBMC *ex vivo*, Table 1).

**Table 1:** Correlations between IDO1 and ARG1 mRNA levels and cytokine and COX2 mRNA levels in PBMCs from HCs and CIS and MS patients. Correlation coefficients (r) are shown; a negative sign indicates an inverse correlation. Bold indicates a statistically significant correlation (*P *<* *0.05)

	PBMC *ex vivo*			PBMC culture (4 h, 37°C)		
	HC *n* = 7	CIS *n* = 11	MS *n* = 8	HC *n* = 14	CIS *n* = 14	MS *n* = 8
IDO1						
TNF	0.18	−0.28	−0.21	**0.72** ***	**0.89** ***	**0.86** **
IL‐1B	0.32	−0.37	0.69	**0.76** **	**0.63** *	**0.79** *
IL‐6	−0.04	0.09	0.12	**0.69** **	**0.66** *	**0.74** *
COX2	nd	nd	nd	**0.79** **	**0.84** ***	**0.79** *
IL‐10	−0.18	−0.33	0.55	**0.60** *	**0.62** *	**0.76** *
TGFB	−0.07	0.33	0.00	**0.86** ***	**0.70** **	0.31
ARG1						
TNF	−0.07	−0.21	−0.55	0.49	0.17	0.19
IL‐1B	0.21	−0.19	0.09	0.44	0.29	0.26
IL‐6	−0.25	−0.30	0.36	0.31	0.31	0.29
COX2	nd	nd	nd	0.52	**0.62** *	0.38
IL‐10	−0.39	0.01	0.50	0.44	0.48	0.45
TGFB	−0.27	**0.64** *	−0.14	**0.58** *	**0.82** ***	0.52

*P <* *0.05; **P <* *0.01; ***P <* *0.001; nd, not done.

### Timing of recent demyelinating/inflammatory events and levels of serum 25(OH)D_3_ do not correlate with gene expression of IDO, ARG and cytokines

To test whether recent demyelination and any associated inflammation led to the differences in catabolic enzyme and cytokine mRNA expression, their levels were tested for correlation with the days to venepuncture since CIS diagnosis or MS diagnosis/relapse. No significant correlations were observed for IDO and ARG; however, a weak positive correlation (*P *=* *0.04) was detected for TNF and IL‐6 mRNA expression and days from CIS diagnosis to venepuncture (Supplementary table 1). As levels of TNF and IL‐6 mRNA in cells of CIS patients were generally low and not different to those of HCs, this result may reflect amounts measured in cells from two or three patients only (Figure 2). As immune cells may activate vitamin D for purposes of regulating catabolic enzyme and cytokine expression,^18^, ^19^ correlations were investigated between serum 25‐hydroxy vitamin D3 [25(OH)D_3_] and IDO, ARG and cytokine mRNA levels in PBMCs. No significant relationships were detected and because 25(OH)D levels change with season suggested no significant seasonal effects on the transcripts measured (Supplementary table 1).

### Alterations in gene expression of IDO, ARG and cytokines do not reflect changes in major cell subsets

As higher IDO and ARG mRNA levels in CIS and MS patients may be because of a greater percentage of a major cell subset in patient PBMCs, correlations were sought between the relative mRNA expression levels of IDO and ARG and the frequency of CD3^+^CD4^+^ and CD3^+^CD8^+^ T cells, CD19^+^CD20^+^ B cells, CD3^−^CD56^+^ NK cells and CD14^++^ monocytes in PBMCs from HCs and CIS and MS patients. These subsets were chosen because they can represent more than 10% of circulating PBMCs in the groups under analysis and have been shown previously to express significant levels of IDO and ARG.^14^ No significant associations were observed with any cell population in the three blood donor groups suggesting the increase in IDO and ARG expression observed in CIS and MS was not linked to elevated numbers of a specific cell subset (data not shown). Furthermore, no relationships were observed between cytokine mRNA levels and a PBMC subset (data not shown).

Correlations between IDO and ARG expression and numerically less frequent cell subsets were next analysed as those cells may not be expressing the detected enzyme mRNA but may regulate levels of expression by other cells. A positive relationship between the proportion of T_reg_ cells and ARG1 and ARG2 mRNA levels was detected in HCs, but this relationship was not observed in CIS or MS patients (Supplementary table 2). The proportion of CD3^−^CD56^hi^CD16^lo^ NK cells (representing approximately 10% NK cells) in PBMCs from CIS patients was associated with greater IDO1 mRNA levels (Supplementary table 2) but is of unknown meaning as this relationship was not detected for the higher IDO1 levels in cells from MS patients (Figure 1). In MS, the proportion of switched memory B cells inversely correlated with IDO1 and IDO2 mRNA expression; however, to our knowledge, a reduction of switched memory B cells has not been reported in MS (Supplementary table 2). These associations were detected, but their meaning remains elusive. No associations were detected with naïve B cells **(**CD19^+^CD20^+^CD27^−^IgD^+^CD38^−^CD24^−/+^), nonswitched memory B cells (CD19^+^CD20^+^CD27^+^IgD^+^), CD3^−^CD56^lo^ CD16^hi^ NK cells, CD14^++^CD16^−^ classical monocytes, CD14^++^ CD16^+^ intermediate monocytes and CD14^+^ CD16^++^ nonclassical monocytes.

There were some significant correlations between cytokine mRNA levels and the numerically minor cell subsets (Supplementary table 2). This may reflect indirect relationships and/or multiple testing and require further investigations before any conclusions can be drawn.

### Phototherapy stabilises ARG1 expression

**Figure 3:**
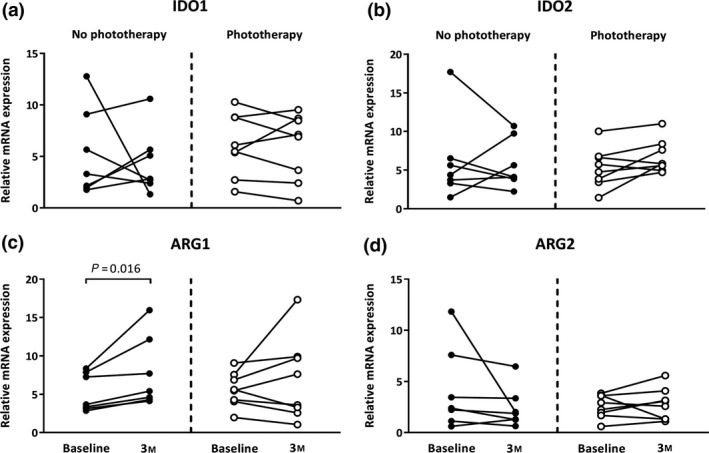
Phototherapy stabilises ARG1 mRNA expression in cells from CIS patients. Changes in (**a**) IDO1, (**b**) IDO2, (**c**) ARG1 and (**d**) ARG2 mRNA expression in PBMCs isolated at baseline and 3 months (3M) from CIS patients in the no phototherapy (*n* = 7, solid circles) and phototherapy (*n* = 8, open circles) groups. Significant differences in expression between baseline and 3M PBMC samples are indicated.

CIS patients (all participants in the PhoCIS trial)^16^, ^17^ donated blood again 3 months after their first venepuncture. There were no differences in the expression of IDO1, IDO2 and ARG2 mRNA in cells from the participants (Figure 3a, b and d). However, for cells from CIS patients not receiving phototherapy, the expression of ARG1 significantly increased at 3 months compared with their baseline sample (Figure 3c). This result supports higher ARG1 expression by PBMCs from those with MS and that the CIS patients studied were on a trajectory for conversion to MS (all CIS patients not receiving phototherapy converted to MS by 12 months of follow‐up).^17^ The increase after 3 months from baseline in ARG1 mRNA levels was not observed in the CIS patients that received phototherapy and suggested that the intervention may have stimulated pathways that stabilise ARG1 expression. There were no associations between mRNA for the catabolic enzymes and the cytokines after 3 months, or changes in cytokine mRNA levels between baseline and 3‐month samples (data not shown).

### IDO1 expression is regulated by extrinsic cues whilst ARG1 expression is intrinsic in MS

**Figure 4:**
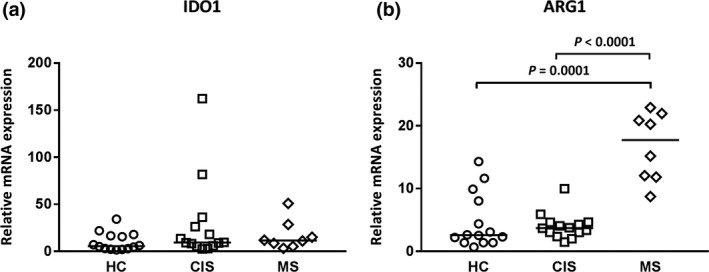
Elevated ARG1, but not IDO1 expression, in PBMCs from MS patients after culture. (**a**) IDO1 and (**b**) ARG1. The median (horizontal bar) and significant differences between expression in PBMCs from HCs (*n* = 14, circles), CIS (*n* = 14, squares) and MS patients (*n* = 8, diamonds) are indicated.

Next, PBMCs were incubated for 4 h in medium containing low maintenance levels of Trp (0.02 mmol L^−1^) and Arg (1.15 mmol L^−1^) but no extracellular activation signals. It was proposed that mRNA levels after 4 h would reflect ongoing endogenous stimulation. The elevated mRNA levels of IDO1 in cells from CIS and MS patients were not detected in their PBMCs after 4 h culture (Figure 4a) and suggested that the increased IDO1 mRNA in cells *ex vivo* was stimulated by extrinsic cues. In contrast, higher ARG1 mRNA expression in cells from MS patients compared with that in cells from HCs was sustained after culture (Figure 4b). This is further demonstrated in Supplementary table 3, where a significant association was observed between ARG1, but not IDO1 mRNA levels, in cells directly *ex vivo* and after culture for 4 h. This result suggests an intrinsic change in PBMCs from MS patients for increased ARG1 expression.

### IDO1 expression is associated with the production of proinflammatory cytokines

**Figure 5:**
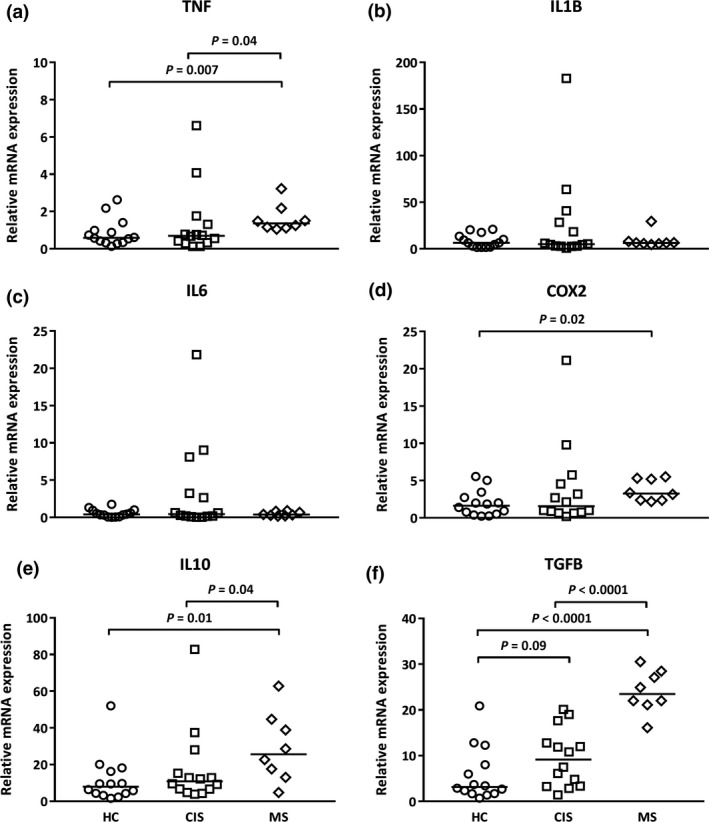
Elevated mRNA expression of pro‐ and anti‐inflammatory cytokines in cultured PBMCs from MS patients. **(a)** TNF,** (b)** IL‐B, **(c)** IL‐6, **(d)** COX2, **(e)** IL‐10 and **(f)** TGFB. The median (horizontal bar) and significant differences between expression in PBMCs from HCs (*n* = 14, circles), CIS (*n* = 14, squares) and MS patients (*n* = 8, diamonds) are indicated.

As no correlations were detected between mRNA levels of cytokine genes and mRNA of IDO and ARG in PBMCs directly *ex vivo*, associations were sought for PBMCs cultured without stimulation for 4 h. The proinflammatory mediators chosen for study were TNF, IL‐1B, IL‐6 and the enzyme, COX2. PBMCs from MS but not CIS patients expressed significantly higher levels of TNF and COX2 (Figure 5a and d). However, of the levels expressed, mRNA levels of TNF, IL‐1B, IL‐6 and COX2 in all groups of individuals studied, including HCs, positively and significantly correlated with IDO1 mRNA levels (columns for PBMC culture, Table 2). This supported the hypothesis of an association between IDO1 and cytokine expression, particularly when more cytokines may be expressed in MS. ARG1 mRNA did not correlate with mRNA of proinflammatory cytokines, only for COX2 mRNA and only in cells from CIS patients (Table 2).

**Table 2:** Cohort demographics shown as median (minimum–maximum)

	HC	CIS	MS
*n*	18	17	8
Age in years	38 (26–61)	36 (26–54)	44 (18–54)
Male/Female	8/10	7/10	3/5
Days to venepuncture from CIS or MS diagnosis by MRI	–	37 (−1 to 119)	6 (0–22)
Serum 25(OH)D_3_ in nmol L^−1^	nd	91.5 (43.7–135.6)	69.9 (40.8–108.2)

nd, not determined.

### IDO1 expression correlates with IL‐10, but both IDO1 and ARG1 correlate with TGFB mRNA in cultured cells from HCs and CIS patients

When mRNA levels for the anti‐inflammatory cytokines, IL‐10 and TGFB, were examined in cultured cells, significantly higher expression was detected for cells from MS patients only (Figure 5e and f). This did not match the pattern detected for PBMCs analysed *ex vivo* (Figure 2d and e), with the pattern of expression of TGFB mRNA being an inverse of that of cells *ex vivo*. For all three blood donor groups, mRNA levels of IL‐10 correlated significantly with IDO1 mRNA levels. For TGFB mRNA, correlations with IDO1 were detected only for HCs and CIS patients (columns for PBMC culture, Table 2). ARG1 mRNA levels associated only with TGFB mRNA in cells from HCs and CIS patients (columns for PBMC culture, Table 2). These correlations suggest that, in the absence of strong extrinsic regulators *in vivo*, the expression of both pro‐ and anti‐inflammatory cytokines and COX2 positively and significantly associated with the expression of IDO in PBMCs. Significant correlations with ARG1 mRNA expression were limited to TGFB.

## Discussion

Greater cellular expression of IDO and ARG has been associated with a homeostatic or normalising response to cellular activation and with degradation of amino acids that would otherwise be available to increase a proinflammatory response. The PBMCs of the MS patients studied had no adverse dampening of the expression of IDO and ARG; such a block may otherwise contribute to disease pathogenesis. There was no correlation in PBMCs *ex vivo* from HCs and patients with CIS and MS between mRNA levels for IDO and ARG, and expression of pro‐ or anti‐inflammatory cytokine genes. However, there were differences in PBMC mRNA levels for the catabolic enzymes and for cytokines (independently of each other) according to patient disease stage. Emphasis was placed on measures of mRNA directly *ex vivo* because accurate measures of the translated protein, particularly cytokines which are not stored intracellularly, require culture of the cells *in vitro*. This study demonstrates the labile nature of cytokine and enzyme mRNA as they can change with cell incubation for 4 h under contrived *in vitro* conditions. This study confirms that mRNA levels best capture direct *ex vivo* expression.

Greater percentages of a major cell type in PBMCs were not found to be responsible for higher mRNA levels of IDO and ARG in cells from CIS and MS patients. It remains possible that single or multiple cell subsets express increased levels of the enzymes. Both decreases^14^ and increases^20^ in ARG1 in monocytes from MS patients have been previously reported.

Dysfunction in the pathway downstream of IDO in Trp metabolism has been implicated in MS pathogenesis,^21^ particularly when IDO‐expressing cells leave the circulation and enter the central nervous system. The kynurenines produced by Trp catabolism may be neuroprotective (kynurenic acid, picolinic acid) or neurotoxic (3‐hydroxykynurenine, quinolinic acid), with dysfunction suggested in MS by greater activity in pathways leading to production of neurotoxic mediators.^22^ With measurement of greater IDO1 mRNA in cells from patients with CIS, the present study suggests greater flux through the kynurenine pathway early in the MS disease course.

Our study suggests that in CIS, lower IL‐10 and TGFB mRNA expression in PBMCs precedes any stimulation of proinflammatory cytokine expression. In cells *ex vivo* from the CIS patients, mRNA levels of the anti‐inflammatory cytokines, IL‐10 and TGFB, were significantly reduced compared with levels in HCs. There was no expression of the proinflammatory cytokines in CIS. It was only in cells from MS patients that significantly higher IL‐6 mRNA levels were detected. In the established MS patients, IL‐10 mRNA levels were also no longer different to those measured in cells from HCs and suggested a shifted balance towards a more inflammatory cytokine transcriptional signature. In a pattern different to that of IL‐10, mRNA levels for TGFB, already low in CIS individuals, were lower again in PBMCs from MS patients. Low levels of expression of tolerance‐inducing TGFB have been reported in experimental autoimmune encephalomyelitis, the murine model of neuroinflammation used to study MS, with increased expression after treatment with the disease modifier, fingolimod.^23^ Also, in PBMCs from patients with established disease, TNF mRNA levels are higher and IL‐10 and TGFB levels are lower during relapse, and this balance is inverted during stable disease.^24^

Cytokine and catabolic enzyme mRNA levels were re‐examined after culture of PBMCs for 4 h, a time frame that is generally longer than the half‐life of mRNA. A 4‐h culture period is also the time popularly used for sensitive detection of cytokines that have accumulated intracellularly after blocking their secretion through the endoplasmic reticulum.^25^ The differing levels of IL‐10 mRNA in cells *ex vivo* were not replicated and suggested that during culture, an important external modulating agent was not present. Further, the potency of the modulating agent was highlighted as cells from MS patients now expressed higher IL‐10 mRNA levels. The pattern of TGFB levels in cells from HCs, and CIS and MS patients was also different, further highlighting the strength of an extrinsic TGFB‐suppressive agent *in vivo* that was not present in culture. When the relationship between cytokine and IDO and ARG expression was reinvestigated after culture, IDO1 and ARG1 mRNA levels in PBMCs *ex vivo* from HCs and CIS patients did not correlate with levels measured after culture. In PBMCs from the MS patients where levels of expression were greater and associations more likely detected, the increased IDO1 mRNA expression was lost but the higher ARG1 mRNA levels were maintained in PBMCs after culture. This suggested that in the cells of MS patients, the higher IDO1 mRNA levels in PBMCs *ex vivo* were extrinsically determined but those of ARG1 mRNA levels were determined by intrinsic signals. For cultured cells from all blood donor groups, IDO1 mRNA significantly correlated with levels of TNF, IL‐1B, IL‐6, COX2, IL‐10 and TGFB mRNA (with the exception of TGFB in PBMCs from MS patients). For cultured cells from HCs and CIS (but not MS patients), ARG1 associated positively only with TGFB mRNA levels; this supported an ARG‐TGFB inter‐relationship with T_reg_ induction (Table 2).^26^ However, in PBMCs from MS patients, this link may be impaired because of ARG1 intrinsic changes described above.

Greater ARG1 expression in MS compared with CIS patients was confirmed when PBMCs from 15 patients with CIS were restudied 3 months after their first blood donation. In cells from those who did not receive phototherapy, the levels of expression of ARG1 increased, a rise not measured in PBMCs from those who underwent an 8‐week course of narrowband UVB phototherapy. As increases in ARG expression have been attributed to inflammatory signals,^27^, ^28^ narrowband UVB phototherapy may have reduced the expression of those signals.

Our study does not support the reduced expression of IDO1 and ARG1 in PBMCs from Argentinian patients with clinically definite, relapsing–remitting MS.^14^ To explain differences in study outcomes, 62% of the Argentinian cohort were taking IFN‐β1α or glatiramer acetate. Our patients may be genetically different, and IDO and ARG expression may vary with activity or duration of MS. In the Argentinian study, culture of the blood cells also altered IDO1 and ARG1 expression. Increased ARG1 expression in PBMCs from MS patients is supported by a study of macrophages from the blood of disease‐modifying treatment (DMT)‐free patients with established MS.^20^ These cells expressed increased ARG1 *in vitro,* and they were more susceptible to activation with an enhanced ability, relative to macrophages from normal people, to skew towards inducible NOS‐ or ARG‐expressing cells in the presence of LPS or IL‐4, respectively.^20^

In summary, IDO1 and ARG2 mRNA levels were higher in cells from CIS patients relative to HCs. However, they had no association with the significantly lower regulatory cytokine mRNA levels in circulating PBMCs from those with CIS. Only after 4 h in culture, IDO1 expression associated with levels of both pro‐ and anti‐inflammatory cytokines, and ARG1 associated with TGFB mRNA, indicating potential catabolic enzyme–cytokine coregulation. This suggested removal of a suppressive regulator rather than intrinsic changes in the cells and highlights the complexity of regulating immune cells in CIS and MS, and the need to study external factors in serum that may have been produced by disease‐associated nonhaematopoietic cells. This study highlights that in patients with CIS and who are not yet diagnosed as MS, there are already changes to circulating haematopoietic cells that contribute to immune cell dysfunction.

## Methods

### Selection of donors of peripheral blood

PBMCs were isolated from 17 individuals with CIS and eight with MS. The CIS patients (with clinical symptoms as detailed previously)^17^ donated blood within 120 days of confirmation of their first demyelinating event by MRI and were at high risk for MS conversion as their MRI satisfied Paty A or Paty B criteria.^16^, ^17^, ^29^ All CIS patients were participants in the PhoCIS trial,^16^, ^17^ with their baseline sample included in the present study. Seven of the eight patients with MS had recent clinical symptoms but their MRI showed a diagnosis of MS by 2010 McDonald criteria; that is, their cerebral and/or spinal cord MRI indicated that they had multiple demyelinating lesions separated in time and space.^30^ Despite subjective recollection of symptoms in past years, only one MS patient had been previously formally diagnosed with MS and that was 7 years before. Blood for PBMC isolation was drawn from that MS patient 20 days after a clinical relapse. At the time of venepuncture, all CIS and MS blood donors had not taken steroids for at least 1 month, or ever received a DMT. PBMCs were also isolated from 18 HCs with no history of autoimmune disease. The demographic data for the groups are displayed in Table 2.

The study was approved by the Bellberry Human Research Ethics Committee (2014‐02‐083) and endorsed by the Human Research Ethics Office of the University of Western Australia (RA/4/1/6796). Informed consent was obtained from all blood donors.

### Narrowband UVB phototherapy intervention

The CIS patients were randomly assigned into two groups as previously described.^16^, ^17^ One group received narrowband UVB phototherapy (wavelengths 311‐312 nm) 3 times per week for 8 weeks (24 exposures in total) to their full body.^16^, ^17^ Blood samples were again collected at 3 months and PBMCs isolated. Baseline and 3‐month paired blood samples were available for enzyme and cytokine mRNA analysis from 15 CIS participants (8 receiving phototherapy and 7 in the no phototherapy (i.e. control) group). The trial was registered with the Australian New Zealand Clinical Trials Registry (ACTRN12614000185662).

### Isolation of PBMCs for direct RNA isolation or cell culture

Peripheral venous blood was collected into lithium heparin tubes (BD Vacutainer Systems, Plymouth, UK) and processed within 1‐2 h. PBMCs were isolated by density gradient centrifugation using Lymphoprep (Axis‐Sheild, Oslo, Norway) and cryopreserved in liquid nitrogen at 5‐10 × 10^6^ cells mL^−1^ in 10% DMSO/FCS (Sigma‐Aldrich, St Louis, MO, USA) until analysis.

Cryopreserved PBMCs were thawed, washed and resuspended at 10^6^ cells mL^−1^ in RPMI 1640 medium (HyClone; GE Health Care Life Sciences, Logan, UT) supplemented with 10% FCS (HyClone), 5 μg mL^−1^ gentamicin, 2 mm l‐glutamine and 50 μm 2‐β‐mercaptoethanol (all from Sigma‐Aldrich). For *ex vivo* PBMC preparation, duplicate samples of 10^6^ cells were pelleted (500 × g, 5 min), then homogenised in 500 μL TRIzol (Life Technologies, Carlsbad, CA) and incubated for 5 min to permit complete dissociation of nucleoproteins. Cell lysates were stored at −80°C until batch processing. For PBMC cultures, 10^6^ cells mL^−1^ were incubated in medium (as above) at 37°C/5% CO₂ for 4 h. PBMCs were then pelleted, homogenised in TRIzol and stored as previously described.

### RNA extraction and cDNA synthesis

To extract total RNA, a modified TRIzol/RNeasy Mini Kit (Qiagen, Hilden, Germany) protocol was utilised. Chloroform (100 μL; Merck Millipore, Darmstadt, Germany) was added to cell lysates, vigorously mixed and incubated for 2 min. The samples were separated by centrifugation (14 000 *g*, 5 min at 4°C) and the aqueous layer containing RNA transferred to a new tube and mixed with an equal volume of 70% ethanol. The solution was transferred to a RNeasy spin column and the protocol continued according to the manufacturer's instructions. RNA was quantitated using a Qubit 3.0 (Thermo Fisher Scientific, Waltham, MA), and the absorbance at 260:280 nm was used as a measure of purity (NanoDrop 2000 Spectrophotometer, Thermo Fisher Scientific). All RNA samples were standardised to a concentration of 25 ng μL^−1^ and reverse‐transcribed to cDNA with the iScript gDNA Clear cDNA Synthesis Kit (Bio‐Rad, Hercules, CA).

### Quantitative real‐time polymerase chain reaction

Synthesised cDNA samples were diluted to 8 ng μL^−1^ (20 ng total RNA in 2.5 μL per reaction) and combined with 2× SsoAdvanced Universal Probes Supermix (Bio‐Rad), RNase‐free water and the following PrimerPCR Probes all fluorescently labelled with FAM (Bio‐Rad). For analysis of cells *ex vivo*, probes were for IDO1, IDO2, ARG1, ARG2, nitric oxide synthase 2 (NOS2), TNF, IL‐1B, IL‐6, IL‐10, transforming growth factor beta 1 (TGFB) and UBE2D2. For analysis of cultured cells, probes were for TNF, IL‐1B, IL‐6, IL‐10, TGFB, cyclooxygenase 2 (COX2), IDO1, ARG1 and UBE2D2. UBE2D2 was used as the housekeeping gene because in a comparison with seven other potential housekeeping genes, it was the most stably expressed gene in cell subsets from HCs and those with relapsing–remitting MS.^31^ All samples were loaded onto a 96‐well plate that contained a nonreverse transcriptase control and a no template control. An aliquot from a large cDNA preparation (frozen as multiple vials) was added to every plate for UBE2D2 measurement and used as a PCR quality control. Levels of PBMC mRNA were determined by RT–PCR performed on a ABI Prism 7900HT sequence detection system (Applied Biosystems, Foster City, CA) with the following cycling conditions: 95°C for 30 s, 40 cycles of amplification at 95°C for 15 s and 60°C for 1 min.

### Calculating relative gene expression

The threshold cycle (*C*_T_) values were determined for all genes and exported from SDS software (Applied Biosystems). The *C*_T_ cut‐off was set to 35 for all genes, and relative gene expression was determined by normalising to the housekeeping gene (Δ*C*_T_ **=* *gene of interest – UBE2D2). Normalised values of duplicate samples were then averaged and inversely transformed (2^(−ΔCT)^). Comparison of the PCR quality controls for 26 plates showed a maximum difference of 0.5 *C*_T_ with a standard deviation of 0.1. All genes were detectable except for NOS2 which was subsequently excluded from analysis. In some samples, IDO2 was below the level of detection and a *C*_T_ value of 35 was assigned for inclusion.

### T cell, B cell, NK cell and monocyte subset analysis, and vitamin D measurement

The frequencies of major (CD4^+^ T cells, CD8^+^ T cells, B cells, NK cells and monocytes) and numerically minor cell subsets (T_reg_ cells, naïve B cells, nonswitched memory B cells, switched memory B cells, CD56^lo^CD16^hi^ NK cells, CD56^hi^CD16^lo^ NK cells, classical monocytes, intermediate monocytes and nonclassical monocytes) were assessed by flow cytometry in freshly isolated PBMCs from HCs and CIS and MS patients as previously reported.^25^, ^32^ Serum levels (25(OH)D_3_) were measured as previously described.^33^

### Statistical analyses

Statistical analyses were performed using SPSS Statistics v25 (IBM Corp., Armonk, NY), and figures were generated in GraphPad Prism v7 (GraphPad Software, La Jolla, CA). Nonparametric tests were conducted to determine statistical significance. Wilcoxon matched pairs tests were used to compare baseline and 3‐month results within groups, and Kruskal–Wallis and Mann–Whitney tests were used to compare results between blood donor groups. Correlation coefficients were calculated with Spearman's tests. *P* values < 0.05 were considered statistically significant.

## References

[1] Pearce EL , Pearce EJ . Metabolic pathways in immune cell activation and quiescence. Immunity 2013; 38: 633–643. 23601682 10.1016/j.immuni.2013.04.005 PMC3654249

[2] Geiger R , Rieckmann JC , Wolf T *et al* L‐arginine modulates T cell metabolism and enhances survival and anti‐tumor activity. Cell 2016; 167: 829–842. 27745970 10.1016/j.cell.2016.09.031 PMC5075284

[3] Mandi Y , Vecsei L . The kynurenine system and immunoregulation. J Neural Transm (Vienna) 2012; 119: 197–209. 21744051 10.1007/s00702-011-0681-y

[4] Mbongue JC , Nicholas DA , Torrez TW , Kim NS , Firek AF , Langridge WH . The role of indoleamine 2, 3‐dioxygenase in immune suppression and autoimmunity. Vaccines (Basel) 2015; 3: 703–729. 26378585 10.3390/vaccines3030703 PMC4586474

[5] Grohmann U , Mondanelli G , Belladonna ML *et al* Amino‐acid sensing and degrading pathways in immune regulation. Cytokine Growth Factor Rev 2017; 35: 37–45. 28545736 10.1016/j.cytogfr.2017.05.004

[6] Fallarino F , Grohmann U , You S *et al* The combined effects of tryptophan starvation and tryptophan catabolites down‐regulate T cell receptor zeta‐chain and induce a regulatory phenotype in naive T cells. J Immunol 2006; 176: 6752–6761. 16709834 10.4049/jimmunol.176.11.6752

[7] Mondanelli GR , Bianchi R , Pallotta MT *et al* A relay pathway between arginine and tryptophan metabolism confers immunosuppressive properties on dendritic cells. Immunity 2017; 46: 233–244. 28214225 10.1016/j.immuni.2017.01.005 PMC5337620

[8] Mellor AL , Lemos H , Huang L . Indoleamine 2,3‐dioxygenase and tolerance: where are we now? Front Immunol 2017; 8: 1360. 29163470 10.3389/fimmu.2017.01360 PMC5663846

[9] Gutierrez‐Vazquez C , Quintana FJ . Regulation of the immune response by the aryl hydrocarbon receptor. Immunity 2018; 48: 19–33. 29343438 10.1016/j.immuni.2017.12.012 PMC5777317

[10] Burrack KS , Morrison TE . The role of myeloid cell activation and arginine metabolism in the pathogenesis of virus‐induced diseases. Front Immunol 2014; 5: 428. 25250029 10.3389/fimmu.2014.00428 PMC4157561

[11] Qualls JE , Murray PJ . Immunometabolism within the tuberculosis granuloma: amino acids, hypoxia, and cellular respiration. Semin Immunopathol 2016; 38: 139–152. 26490974 10.1007/s00281-015-0534-0 PMC4779414

[12] Morris SM Jr . Arginine: master and commander in innate immune responses. Sci Signal 2010; 3: pe27. 20716762 10.1126/scisignal.3135pe27

[13] McGovern N , Shin A , Low G *et al* Human fetal dendritic cells promote prenatal T‐cell immune suppression through arginase‐2. Nature 2017; 546: 662–666. 28614294 10.1038/nature22795 PMC6588541

[14] Negrotto L , Correale J . Amino acid catabolism in multiple sclerosis affects immune homeostasis. J Immunol 2017; 198: 1900–1909. 28130499 10.4049/jimmunol.1601139

[15] Rostami A , Ciric B . Role of Th17 cells in the pathogenesis of CNS inflammatory demyelination. J Neurol Sci 2013; 333: 76–87. 23578791 10.1016/j.jns.2013.03.002 PMC3726569

[16] Hart PH , Lucas RM , Booth DR *et al* Narrowband UVB phototherapy for Clinically Isolated Syndrome: a trial to deliver the benefits of vitamin D and other UVB‐induced molecules. Front Immunol 2017; 8: 3. 28167940 10.3389/fimmu.2017.00003 PMC5256075

[17] Hart PH , Jones AP , Trend S *et al* A randomised controlled clinical trial of narrowband UVB phototherapy for Clinically Isolated Syndrome: the PhoCIS study. Mult Scler J Exp Transl Clin 2018; 4: 2055217318773112. 29780610 10.1177/2055217318773112 PMC5954316

[18] Baeke F , Takiishi T , Korf H , Gysemans C , Mathieu C . Vitamin D: modulator of the immune system. Curr Opin Pharmacol 2010; 10: 482–496. 20427238 10.1016/j.coph.2010.04.001

[19] Lucas RM , Gorman S , Geldenhuys S , Hart PH . Vitamin D and immunity. F1000Prime Rep 2014; 6: 118. 25580272 10.12703/P6-118 PMC4251419

[20] Christophi GP , Panos M , Hudson CA *et al* Macrophages of multiple sclerosis patients display deficient SHP‐1 expression and enhanced inflammatory pathways. Lab Invest 2009; 89: 742–759. 19398961 10.1038/labinvest.2009.32 PMC2725397

[21] Lovelace MD , Varney B , Sundaram G *et al* Current evidence for a role of the kynurenine pathway of tryptophan metabolism in multiple sclerosis. Front Immunol 2016; 7: 246. 27540379 10.3389/fimmu.2016.00246 PMC4972824

[22] Lim CK , Bilgin A , Lovejoy DB *et al* Kynurenine pathway metabolomics predicts and provides mechanistic insight into multiple sclerosis progression. Sci Rep 2017; 7: 41473. 28155867 10.1038/srep41473 PMC5290739

[23] Hou H , Cao R , Miao J *et al* Fingolimod ameliorates the development of experimental autoimmune encephalomyelitis by inhibiting Akt‐mTOR axis in mice. Int Immunopharmacol 2016; 30: 171–178. 26632437 10.1016/j.intimp.2015.11.024

[24] Rieckmann P , Albrecht M , Kitze B *et al* Tumor necrosis factor‐alpha messenger RNA expression in patients with relapsing‐remitting multiple sclerosis is associated with disease activity. Ann Neurol 1995; 37: 82–88. 7818262 10.1002/ana.410370115

[25] Jones AP , Trend S , Byrne SN *et al* Altered regulatory T‐cell fractions and Helios expression in clinically isolated syndrome: clues to the development of multiple sclerosis. Clin Transl Immunology 2017; 6: e143. 28690849 10.1038/cti.2017.18 PMC5493587

[26] Horwitz DA , Zheng SG , Gray JD . The role of the combination of IL‐2 and TGF‐beta or IL‐10 in the generation and function of CD4+ CD25+ and CD8+ regulatory T cell subsets. J Leukoc Biol 2003; 74: 471–478. 14519757 10.1189/jlb.0503228 PMC7166542

[27] Bronte V , Zanovello P . Regulation of immune responses by L‐arginine metabolism. Nat Rev Immunol 2005; 5: 641–654. 16056256 10.1038/nri1668

[28] McGaha TL , Huang L , Lemos H *et al* Amino acid catabolism: a pivotal regulator of innate and adaptive immunity. Immunol Rev 2012; 249: 135–157. 22889220 10.1111/j.1600-065X.2012.01149.x PMC4384693

[29] Paty DW , Oger JJ , Kastrukoff LF *et al* MRI in the diagnosis of MS: a prospective study with comparison of clinical evaluation, evoked potentials, oligoclonal banding, and CT. Neurology 1988; 38: 180–185. 3340277 10.1212/wnl.38.2.180

[30] Polman CH , Reingold SC , Banwell B *et al* Diagnostic criteria for multiple sclerosis: 2010 revisions to the McDonald criteria. Ann Neurol 2011; 69: 292–302. 21387374 10.1002/ana.22366 PMC3084507

[31] Oturai DB , Sondergaard HB , Bornsen L , Sellebjerg F , Christensen JR . Identification of suitable reference genes for peripheral blood mononuclear cell subset studies in multiple sclerosis. Scand J Immunol 2016; 83: 72–80. 26395032 10.1111/sji.12391

[32] Trend S , Jones AP , Geldenhuys S *et al* Evolving identification of blood cells associated with Clinically Isolated Syndrome: Importance of time since clinical presentation and diagnostic MRI. Int J Mol Sci 2017; 18: 1277. 10.3390/ijms18061277 PMC5486099 28617321

[33] Clarke MW , Tuckey RC , Gorman S , Holt B , Hart PH . Optimized 25‐hydroxyvitamin D analysis using liquid–liquid extraction with 2D separation with LC/MS/MS detection, provides superior precision compared to conventional assays. Metabolomics 2013; 9: 1031–1040.

