# Supplementary material for: Tryptophan and arginine catabolic enzymes and regulatory cytokines in clinically isolated syndrome and multiple sclerosis
Source: Clin Transl Immunology. 2018 Aug 16;7(8):e1037. doi: 10.1002/cti2.1037 (PMC6095938; doi:10.1002/cti2.1037)
Supplement: Supplementary file 1 [file CTI2-7-e1037-s001.pdf]

Supplemetary Tables. Cha et al.

Table 1. Correlations between PBMC catabolic enzymes and cytokine expression (columns) and (a) days from diagnosis, and (b) serum 25(OH)D_3 levels, in CIS patients; and (c) days from relapse, and (d) serum 25(OH)D_3 levels, in MS patients.

	Statistic	IDO1	IDO2	ARG1	ARG2	TNF	IL1B	IL6	IL10	TGFB
**CIS patients**										
**(a) Days from diagnosis to venepuncture**	Correlation coefficient (r)	0.03	-0.30	0.32	0.38	**0.63**	0.55	**0.62**	-0.10	0.58
	P value	0.91	0.25	0.21	0.14	**0.04**	0.08	**0.04**	0.76	0.06
	n	17	17	17	17	11	11	11	11	11
**(b) Serum 25(OH)D_3**	Correlation coefficient (r)	0.20	-0.07	0.25	0.24	0.06	-0.04	-0.12	0.05	0.42
	P value	0.45	0.79	0.33	0.35	0.85	0.89	0.73	0.88	0.20
	n	17	17	17	17	11	11	11	11	11
**MS patients**										
**(c) Days from relapse to venepuncture**	Correlation coefficient (r)	0.28	0.37	0.07	-0.06	0.07	-0.06	-0.30	0.32	0.37
	P value	0.51	0.37	0.87	0.89	0.87	0.89	0.47	0.43	0.37
	n	8	8	8	8	8	8	8	8	8
**(d) Serum 25(OH)D_3**	Correlation coefficient (r)	0.21	0.21	0.12	0.26	-0.43	0.31	0.62	0.52	-0.60
	P value	0.61	0.61	0.78	0.53	0.29	0.46	0.10	0.18	0.12
	n	8	8	8	8	8	8	8	8	8

Shading indicates statistically significant correlations

Table 2. **Significant correlations between PBMC expression of IDO, ARG and cytokine mRNA and frequencies of numerically minor cell subpopulations in (a) HCs, (b) CIS patients, and (c) MS patients.**

	% in PBMC #	Statistic	IDO1	IDO2	ARG1	ARG2	TNF	IL1B	IL6	IL10	TGFB
**(a) HCs**											
**T_{reg} cells** ($CD3^+CD4^+FoxP3^+$ $CXCR5^-$)	2.0-3.7	Correlation coefficient (r)	0.54	0.41	**0.69***	**0.72***	-1.00	-1.00	-1.00	-1.00	-1.00
		n	11	11	11	11	2	2	2	2	2
**Naïve B cells (**$CD19^+CD20^+CD27^-$ $IgD^+CD38^-CD24^{-/+}$)	0.5-9.3	Correlation coefficient (r)	0.05	0.33	-0.13	-0.13	-0.21	0.21	0.75	**0.93****	-0.07
		n	18	18	18	18	7	7	7	7	7
**Non-classical monocytes** ($CD14^+$ $CD16^{++}$)	0.6-3.9	Correlation coefficient (r)	-0.22	0.24	-0.29	-0.05	0.54	**0.79***	0.29	-0.43	-0.11
		n	18	18	18	18	7	7	7	7	7
**(b) CIS patients**											
**T_{reg} cells** ($CD3^+CD4^+FoxP3^+$ $CXCR5^-$)	1.5-4.6	Correlation coefficient (r)	-0.20	-0.33	0.41	-0.12	0.53	**0.70***	0.50	-0.15	0.33
		n	14	14	14	14	11	11	11	11	11
**$CD56^{hi}$ $CD16^{lo}$ NK cells** ($CD3^-$)	0.1-1.0	Correlation coefficient (r)	**0.58***	0.40	-0.09	-0.42	-0.49	**-0.67***	-0.39	0.23	-0.09
		n	17	17	17	17	11	11	11	11	11
**(c) MS patients**											
**Switched memory B cells** ($CD19^+CD20^+$ $CD27^+IgD^-$)	0.02-2.2	Correlation coefficient (r)	**-0.81***	**-0.71***	-0.12	0.1	-0.07	-0.55	-0.14	-0.55	-0.21
		n	8	8	8	8	8	8	8	8	8
**Intermediate monocytes** ($CD14^{++}$ $CD16^+$)	0.2-1.5	Correlation coefficient (r)	**0.76***	0.67	0.24	0.05	-0.52	0.69	0.24	**0.74***	-0.45
		n	8	8	8	8	8	8	8	8	8

* $P < 0.05$ ** $P < 0.01$; shadings indicate statistically significant correlations

Frequencies of PBMC subpopulations displayed as minimum- maximum; shading indicates statistically significant correlations

Table 3. Correlations between the expression of IDO1 and ARG1 in PBMCs *ex vivo* and after culture

	Statistic	IDO1 mRNA (PBMC culture)	ARG1 mRNA (PBMC culture)
**HCs (n=13)**			
**IDO1 mRNA (*ex vivo*)**	Correlation coefficient (r)	-0.10	0.18
	P value	0.74	0.55
**ARG1 mRNA (*ex vivo*)**	Correlation coefficient (r)	-0.27	0.23
	P value	0.38	0.45
**CIS patients (n=12)**			
**IDO1 mRNA (*ex vivo*)**	Correlation coefficient (r)	-0.01	0.51
	P value	0.98	0.09
**ARG1 mRNA (*ex vivo*)**	Correlation coefficient (r)	0.17	0.16
	P value	0.60	0.62
**MS patients (n=8)**			
**IDO1 mRNA (*ex vivo*)**	Correlation coefficient (r)	0.14	0.07
	P value	0.74	0.87
**ARG1 mRNA (*ex vivo*)**	Correlation coefficient (r)	0.05	**0.76**
	P value	0.91	**0.03**

Shading indicates a statistically significant correlation
